# Lack of cathelicidin processing in Papillon-Lefèvre syndrome patients reveals essential role of LL-37 in periodontal homeostasis

**DOI:** 10.1186/s13023-014-0148-y

**Published:** 2014-09-27

**Authors:** Sigrun Eick, Magdalena Puklo, Karina Adamowicz, Tomasz Kantyka, Pieter Hiemstra, Henning Stennicke, Arndt Guentsch, Beate Schacher, Peter Eickholz, Jan Potempa

**Affiliations:** Department of Periodontology, Laboratory of Oral Microbiology, School of Dental Medicine, University of Bern, Bern, Switzerland; Department of Microbiology, Faculty of Biochemistry, Biophysics and Biotechnology, Jagiellonian University, Krakow, 30-386 Poland; Department of Pulmonology, Leiden University Medical Center, Leiden, The Netherlands; Haemostasis Biology, Novo Nordisk A/S, Maaloev, Denmark; Clinic of Prosthetic Dentistry, Center for Dentistry, Jena University Hospital, Jena, Germany; Department of Periodontology, Center for Dental, Oral, and Maxillofacial Medicine (Carolinum), Johann Wolfgang Goethe–University Frankfurt am Main, Frankfurt am Main, Germany; Center of Oral Health and Systemic Diseases, University of Louisville School of Dentistry, Louisville, KY 40202 USA

**Keywords:** Papillon-Lefèvre syndrome, Periodontitis, Cathepsin C, Proteinase 3, Cathelicidin LL-37

## Abstract

**Background:**

Loss-of-function point mutations in the cathepsin C gene are the underlying genetic event in patients with Papillon-Lefèvre syndrome (PLS). PLS neutrophils lack serine protease activity essential for cathelicidin LL-37 generation from hCAP18 precursor.

**Aim:**

We hypothesized that a local deficiency of LL-37 in the infected periodontium is mainly responsible for one of the clinical hallmark of PLS: severe periodontitis already in early childhood.

**Methods:**

To confirm this effect, we compared the level of neutrophil-derived enzymes and antimicrobial peptides in gingival crevicular fluid (GCF) and saliva from PLS, aggressive and chronic periodontitis patients.

**Results:**

Although neutrophil numbers in GCF were present at the same level in all periodontitis groups, LL-37 was totally absent in GCF from PLS patients despite the large amounts of its precursor, hCAP18. The absence of LL-37 in PLS patients coincided with the deficiency of both cathepsin C and protease 3 activities. The presence of other neutrophilic anti-microbial peptides in GCF from PLS patients, such as alpha-defensins, were comparable to that found in chronic periodontitis. In PLS microbial analysis revealed a high prevalence of *Aggregatibacter actinomycetemcomitans* infection. Most strains were susceptible to killing by LL-37.

**Conclusions:**

Collectively, these findings imply that the lack of protease 3 activation by dysfunctional cathepsin C in PLS patients leads to the deficit of antimicrobial and immunomodulatory functions of LL-37 in the gingiva, allowing for infection with *A. actinomycetemcomitans* and the development of severe periodontal disease.

**Electronic supplementary material:**

The online version of this article (doi:10.1186/s13023-014-0148-y) contains supplementary material, which is available to authorized users.

## Background

Papillon-Lefèvre syndrome (PLS), a rare autosomal recessive disease, with an incidence of 1–4 cases/million people is characterized by palmar and plantar hyperkeratosis and severe periodontitis affecting primary and permanent teeth leading to early loss of primary and permanent teeth [1,2]. This periodontitis is classified as a periodontitis as a manifestation of systemic diseases associated with genetic disorders [3]. PLS is caused by lost-of-function mutations in the cathepsin C (CTSC) gene [4,5]. To date, more than 50 different mutations have been identified and since cathepsin C activity is essential for activation of neutrophil elastase, cathepsin G, protease 3 and neutrophil serine protease 4 [6,7], PLS neutrophils show no or severely reduced activity of these four enzymes [8]. Nevertheless, despite this deficiency, only rarely recurrent invasive bacterial infections, e.g. a pyogenic liver abscess are reported [1].

Periodontitis in general is characterised by inflammation of the supporting tissues surrounding the teeth and is one of the most prevalent inflammatory diseases in humans. The prevalence of severe periodontitis increases with age, ranging from 1% in young individuals to about 30% in the older population [9]. Disease is initiated by colonization of certain bacterial species which may change via immunomodulation a symbiotic microbiota into a dysbiotic one, e.g. recently *Porphyromonas gingival*is was postulated to be a “keystone” pathogen [10]. In aggressive periodontitis, a form starting normally in adolescence, *Aggregatibacter actinomycetemcomitans* is highly prevalent, its virulence was mainly equated to the production of a leukotoxin [11].

Polymorphonuclear neutrophils (PMNs) play a major role in immune defence against bacteria. Three serine proteases, proteinase 3 (PR3), neutrophil elastase (NE) and cathepsin G (CTSG), which are components of the neutrophil azurophilic granules, participate in the intracellular killing of phagocytosed pathogens [6]. Further, neutrophils are the most abundant source of the antimicrobial peptides α-defensins 1–4 (human neutrophil peptides 1–4, HNPs 1–4) and hCAP18/LL-37. HNP1-4 are synthesized as prepro forms and stored in a fully processed, biologically active form in primary granules in PMNs [12].

The sole human cathelicidin (hCAP18/LL-37) is encoded by the CAMP gene, and encompasses two distinct domains. The N-terminal, “cathelin-like” domain is structurally conserved amongst vertebrates, which is in stark contrast to the highly diverse antimicrobial peptide constituting the C-terminal domain [13]. The human cathelicidin is highly expressed in the myeloid-linage of bone marrow cells, and also in many types of epithelial cells [14]. In neutrophils hCAP18/LL-37 is stored in specific (secondary) granules as a biologically inactive precursor. During phagocytosis, or in otherwise stimulated neutrophils, bactericidal peptide LL-37 is released from hCAP18/LL-37 by limited proteolysis, which is exerted by PR 3 [15].

There has been reported a consistent association of severe periodontitis with a number of syndromic diseases, especially with those involving neutrophil disorders such as deficient numbers of polymorphonuclear leukocytes or aberrant neutrophil function [2]. *E.g*., patients with Kostmann syndrome have a lack of bactericidal peptide LL-37 [16], other severe congenital neutropenia types affect neutrophil elastase [17]. Recently it was shown that exocytosed material of peripheral blood PMNs of the PLS patients contained abundant hCAP-18 but low levels of LL-37 [18].

Here, we postulate that lack of functional cathepsin C in PLS patients is associated with the absence of functional LL-37 in the gingival region. We further hypothesize that the lack of LL-37 in periodontal tissue is a pivotal factor in the development of severe periodontitis in PLS patients. Towards this aim, levels of hCAP18, LL-37 and neutrophil defensins in gingival crevicular fluid, saliva and peripheral neutrophils of PLS patients were quantified and correlated to LL-37 susceptibility of clinical strains of the causative organism, *Aggregatibacter actinomycetemcomitans*. Cumulatively, our results strongly suggest that antimicrobial and immunomodulatory functions of LL-37 are essential for homeostasis of the periodontium.

## Material and methods

### Subjects

A total of 11 PLS patients (two females) were examined at the Department of Periodontology, Center for Dental, Oral, and Maxillofacial Medicine, Goethe-University Frankfurt/Main. Fasting venous blood and saliva samples were collected from all 11 patients but gingival crevicular fluid only from eight individuals (two were edentulous, one sample failed collection) (Additional file 1: Table S1) [19-24]. Blood was collected from the antecubital fossa (lithium heparin tube, Monovette, Sarstedt AG, Nümbrecht, Germany) from PLS patients and PMNs were isolated using dextran sedimentation followed by hypotonic lysis of erythrocytes. Then cells were resuspended with Hanks balanced salt solution (HBSS) to a density of 3.3*10^6^/mL. PMNs from three healthy subjects prepared in the same way were used as positive controls.

A cohort of patients attending the Center for Dental, Oral and Maxillary Medicine at the University Hospital of Jena was recruited for this study. The subjects included seven patients with aggressive periodontitis (AP; mean age 30.9 years) and 12 with chronic periodontitis (CP; mean age 56.3 years). The patients were diagnosed according to recommendation by the American Academy of Periodontology [3]. Severe periodontitis was diagnosed as an attachment loss of ≥5 mm at a minimum of five sites, in different quadrants, after receiving initial therapy. Nine periodontally healthy subjects were included as a control group (con; mean age 32.2 years). Subjects were free of systemic diseases, and had at least 20 teeth in occlusion. Less than 35% of the patients were active smokers. Individuals who had received systematic periodontal treatment in the preceding year, those who had taken antibiotics within the previous 3 months, or those who were pregnant or nursing were excluded from this study. Clinical examinations included plaque index as a measure for oral hygiene, bleeding on probing (BoP) as a common used index associated with inflammation, probing depths and attachment loss at six sites per tooth. Furthermore, the plaque index had to be less than 0.35 to be selected for the study.

The study protocol was approved by the Ethics Committees of the Universities of Jena (#2030-05/07) and Frankfurt (#31/05), Germany. All participants gave their informed consent.

### Sampling of saliva and crevicular fluid

From all subjects included in the study, saliva and gingival crevicular fluid (GCF) samples (only dentate individuals) were collected in the morning, 2–3 h after breakfast. Whole saliva samples were collected using a sterile glass funnel into weighed 10 mL sterile polypropylene containers for 10 minutes. No oral stimulus was permitted for 120 minutes prior to collection to exclude any influence of mastication or foods. The seated patients collected the saliva over the period and pooled the saliva in the bottom of the mouth and drained to a collection tube when necessary.

Crevicular washes were obtained using a previously described method [25]. The sites to be sampled were isolated with cotton rolls and gently air-dried. A tip was carefully inserted into the crevice at a level of approximately 1 mm below the gingival margin. In each case, seven sequential washes with 10 μL phosphate-buffered saline were performed using a micropipette. The GCF was obtained as a pooled sample from the deepest site in each quadrant, and transferred into an Eppendorf tube. After this, samples were immediately frozen and kept at −20°C until analyzed.

### Microbiota

DNA was extracted from 5 μL of GCF washing using the Genomic Mini Kit (A&A Biotechnology, Gdynia, Poland) according to the manufacturer’s recommendations. Real-time PCR for determining the counts of *A. actinomycetemcomitans, P. gingivalis*, *P. intermedia, T. forsythia* and *T. denticola* was carried out as described recently [26].

### ELISAs

MPO and α-defensin were detected in GCF and saliva samples using Human MPO and Human HNP1–3 (neutrophil defensins) ELISA test kits, respectively, according to manufacturer’s protocol. Both kits were obtained from HyCult Biotechnology (Uden, The Netherlands). Samples were diluted 10-100-fold (saliva), and 10,000-fold (GCF), in PBS and plasma dilution buffer, respectively, for MPO and defensins determination.

### Enzyme activities

Enzyme activities of CTSC and the neutrophil serine proteases NE and PR3 were determined in GCF, saliva and neutrophil lysates from control subjects and PLS patients. Cell lysates were obtained by mixing the neutrophil suspension at a 1:1 ratio with 0.1% hexadecyltrimethyl ammonium bromide (CTAB) followed by incubation at 37°C for 15 min.

The CTSC activity was assayed using H-glycyl-L-arginine-7-amido-4-methylcoumarin (H-Gly-Arg-AMC) (Bachem, Weil, Germany) as a substrate at 500 μM final concentration of 25 mM 2-(N-morpholino)ethanesulfonic acid (MES, Sigma, Munich, Germany), 50 mM NaCl, and 5 mM dithiothreitol (DTT) at pH 6.0. The enzymatic substrate turnover was monitored as the increase of fluorescence (excitation and emission wavelengths at 380 nm and 460 nm, respectively) for 60 min using a Spectramax GEMINI XS (Molecular Devices Corp., Sunnyvale, CA, USA).

The NE activity was determined by measuring the rate of release of *p*-nitroanilide (*p*Na) from *N*-methoxysuccinyl-Ala-Ala-Pro-Val-*p*-nitroanilide (MeSuc-AAPV-*p*NA) used as substrate (Sigma, Munich, Germany). The assay was performed in total volume of 150 μL with 0.75 mM final substrate concentration in 50 mM Tris–HCl, pH 7.5. The rate of *p*NA released was recorded at 405 nm using a Spectromax 250 (Molecular Devices Corp., Sunnyvale, USA) for 30 min.

PR3 activity was determined using Abz-GVADnVADYQ-Y(N0_2_)-D (nV, norvaline) as a substrate at final concentration of 50 μM in 0.1 M Tris–HCl, 5 mM EDTA, 0.15 M NaCl, 0.05% Tween-20, 5% dimethylforamide, pH 7.5. Substrate hydrolysis was measured as an increase of fluorescence at λ_ex_ = 320 nm and λ_em_ = 420 nm for 3 h at 37°C using a Spectramax GEMINI XS.

The activity of CTSC, NE and PR3 in GCF and saliva was calculated as a percentage of activities of individual proteases in lysates of healthy control neutrophils set as 100%.

### Western blot of LL-37

Semiquantitative Western blot analysis of LL-37 was performed as described recently [27]. GCF and saliva samples were diluted 4 times with sample buffer (0.125 mM Tris–HCl, 20% glycerol, 4% SDS), and resolved by SDS-PAGE (16% peptide gel (49.5%T/6%C)) using the Tris-Tricine discontinuous buffer system [28]. LL-37 was synthesized on an Applied Biosystems model 433A synthesizer, and purified by preparative reversed-phase high-performance liquid chromatography (HPLC) [29]. It was used at a concentration of 20 ng/mL (6 ng/well = 4 μg/mL) as a standard. Electrophoresed gels were electroblotted (Trans-Blot Semi-Dry; Bio-Rad) onto polyvinylidene difluoride (PVDF) membranes (Amersham-Pharmacia Co., Uppsala, Sweden). Nonspecific binding sites on the membranes were blocked overnight in 5% skimmed milk (Difco) and immunoblotted. The blots were probed with monoclonal mouse antibodies against LL-37 (clone 1.1C12, 42) and goat anti-mouse IgG horse-raddish peroxidase conjugated antibodies (Sigma). Immunoreactive peptides were detected with ECL Plus (Amersham-Pharmacia Co.) according to manufacturer’s protocol, before membranes were exposed to X-ray films (Kodak, Rochester; NY; USA).

### Determination of antimicrobial activity against Aggregatibacter actinomycetemcomitans

Suspensions of several strains of *A. actinomycetemcomitans* (ATCC 33844 and six clinical isolates) were preincubated with different concentrations of LL-37 for 1 h at 37°C. Strains incubated in the same conditions but without the peptide constituted the control of survival (100% survival). After incubation bacteria were suspended and plated on blood agar plates. Colony forming units were counted after 4 days of cultivation in anaerobic atmosphere.

### Data analysis

All data were entered into the SPSS 21.0 (SPSS Inc, Chicago, IL, USA) program, and were analyzed using the Kruskal-Wallis test. Mann–Whitney *U*-test was used for comparisons with PLS and periodontal healthy subjects each. In GCF, correlations using Spearman test were determined in PLS group and the subjects without known genetic disorders. The level of significance was set to p < 0.05.

## Results

### PLS patients

**Figure 1:**
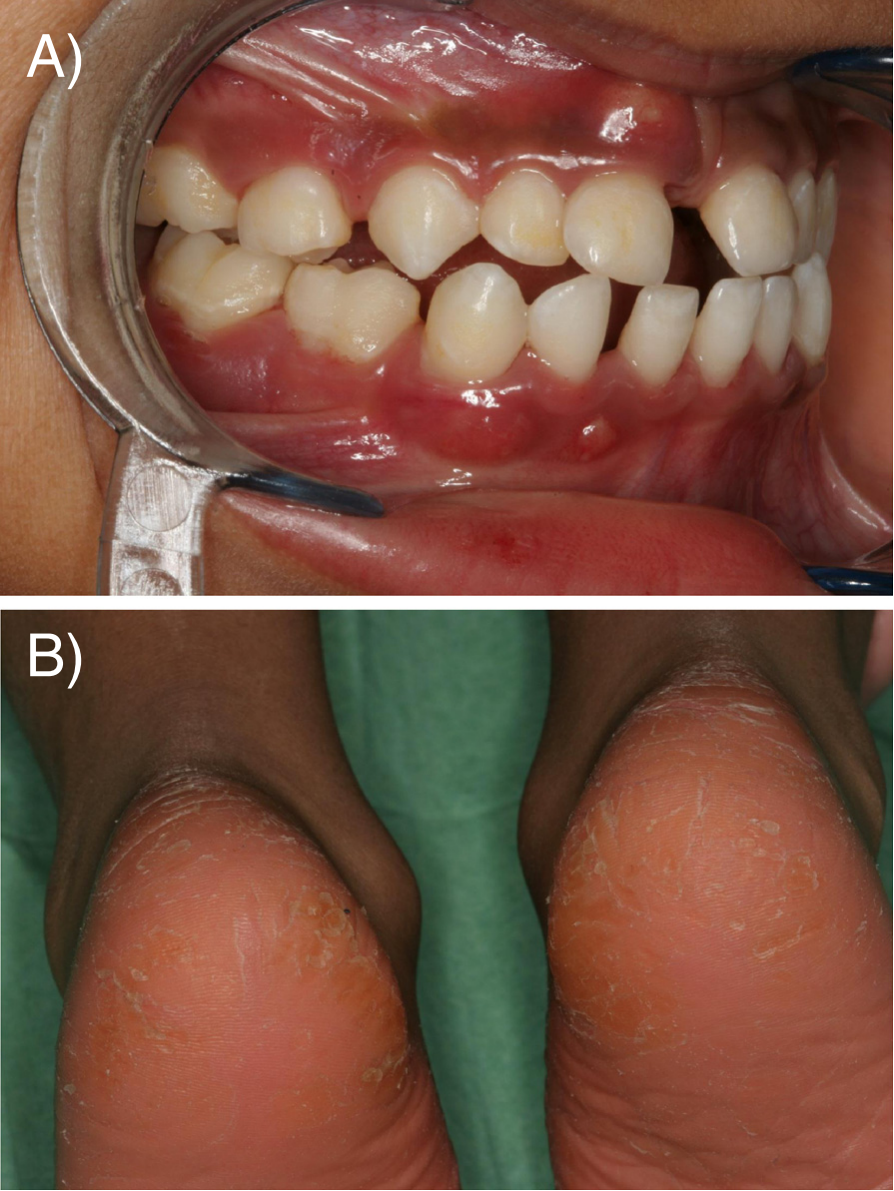
**Papillon Lefèvre syndrome patient.** Clinical view of a Papillon-Lefèvre syndrome patient at 5 years age (#3, Table 1) with typical symptoms: severe periodontitis with a periodontal abscess at the upper central incisor 51 **(A)** and plantar hyperkeratosis **(B)**.

The characteristics, including year of birth, ethnicity, gender, phenotype, the presence of *A. actinomycetemcomitans* in subgingival plaque before treatment and mutation in the CTSC gene (nucleotide, exon, affected amino acid residue) of the 11 PLS patients are summarized in Additional file 1: Table S1). *A. actinomycetemcomitans* was detected in all 9 microbiologically characterized samples before periodontal treatment. Ten out of 11 patients exhibited typical skin lesions. Clinical view of one of the patients is presented in Figure 1. In one patient onset of periodontitis was late (at the age of 22) but, nevertheless, led to edentulism (Additional file 1: Table S1). CTSC, NE and PR3 activity was measured in peripheral blood neutrophils of PLS patients. The CTSC activity was absent in neutrophils from nine patients while in two patients it was detected at the low level (up to 10% of the average activity of three matched healthy controls). In none of neutrophil lysates from PLS patients the PR3 activity was measurable. Low NE activity (at 2.6% of controls) was detected only in one patient with the highest CTSC activity (at 10% of control neutrophils).

For the purpose of this study GCF sampled from eight of the nine dentate patients has been analysed again for bacteria present there. The results were compared to simultaneously performed analysis of samples derived from patients with chronic and aggressive periodontitis, as well as healthy controls. Significantly, despite intensive treatment *A. actinomycetemcomitans* was still detected in GCF from four out of the eight PLS patients at the similar prevalence as in GCF from non-treated aggressive periodontitis. On the other hand, the prevalence of *P. gingivalis* and *T. denticola* was much higher in chronic periodontitis than in PLS patients and the aggressive form of the disease (Table 1). No periodontal pathogens were found in healthy controls.

**Table 1:** **Prevalence of the periodontopathogens** ***Aggregatibacter actinomycetemcomitans*** **,** ***Porphyromonas gingivalis*** **,** ***Tannerella forsythia, Treponema denticola*** **and** ***Prevotella intermedia*** **in gingival crevicular fluid obtained from PLS patients (at the time of the study), periodontitis patients as well as healthy controls**

**Species**	**PLS (n = 8)**	**Aggressive periodontititis (n = 7)**	**Chronic periodontitis (n = 12)**	**Periodontally healthy subjects (n = 9)**
***A. actinomycetemcomitans***	4 (50%)	4 (57%)	4 (33%)	0 (0%)
***P. gingivalis***	0 (0%)	4 (57%)	10 (83%)	0 (0%)
***T. denticola***	1 (13%)	3 (43%)	7 (58%)	0 (0%)
***T. forsythia***	3 (38%)	4 (57%)	11 (92%)	0 (0%)
***P. intermedia***	1 (13%)	2 (29%)	6 (50%)	0 (0%)

### MPO as an equivalent for neutrophil numbers

**Figure 2:**
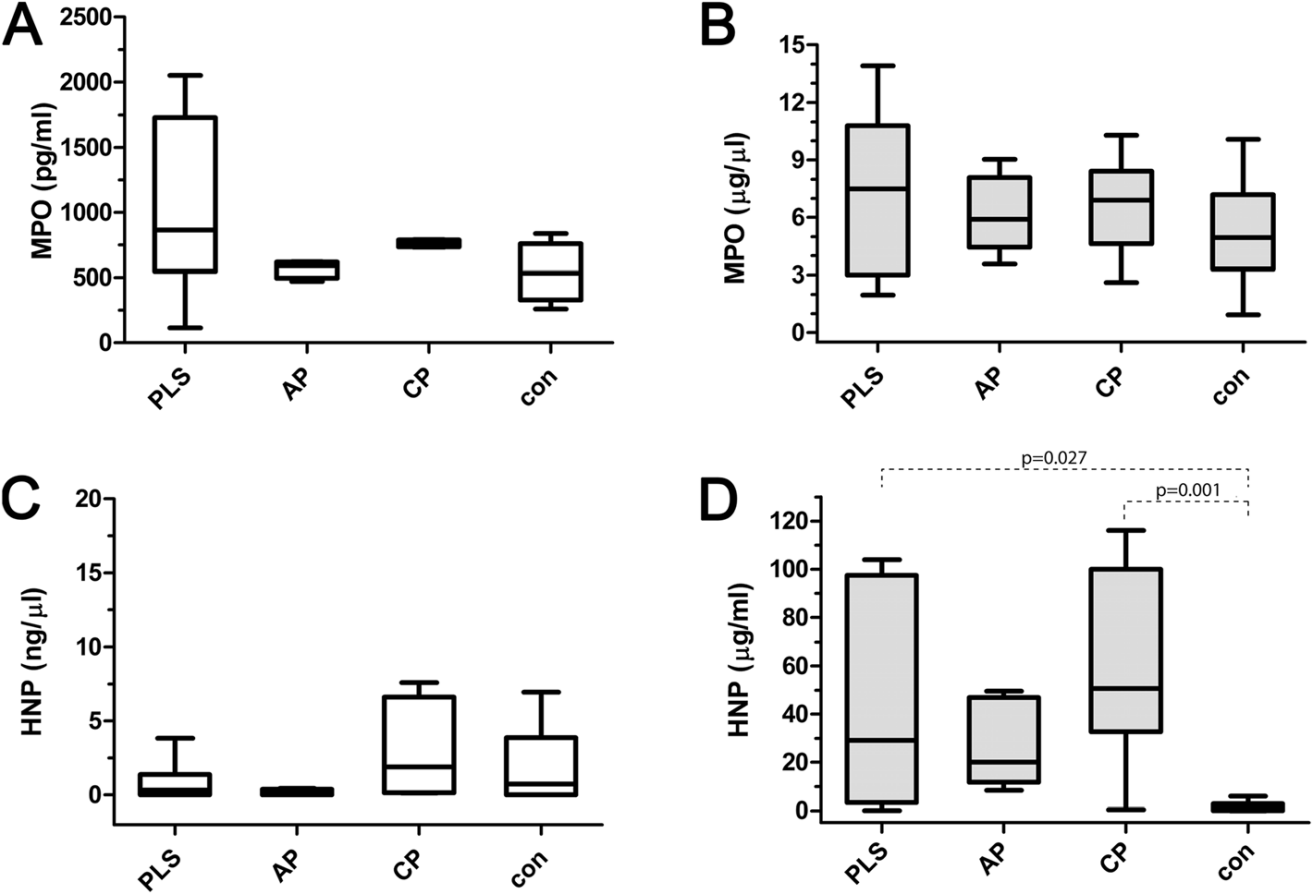
**Levels of myeloperoxidase and α-defensins in saliva and gingival crevicular fluid.** Levels of myeloperoxidase (MPO) **(A and** **B)** and α-defensins (HNP1-3) **(C and** **D)** in saliva **(A and** **C)** and gingival crevicular fluid **(B and** **D)** obtained from Papillon-Lefèvre patients (PLS), aggressive periodontitis patients (AP), chronic periodontitis patients (CP) and healthy controls (con). The results present median, and 25^th^ and 75^th^ percentiles.

Myeloperoxidase (MPO) occurs in high amounts exclusively in neutrophils and its concentration in pathophysiological fluids reflects neutrophil level [27]. Therefore we determined MPO concentration to illustrate relative abundance of neutrophils in our samples. In saliva of PLS patients the median concentration of MPO was determined at 864 pg/ml (range 116 – 2050 pg/ml), thus higher, although without statistical significance, than in other subject groups (CP: 747 pg/ml, range 522–790 pg/ml; AP: 591 pg/ml, range 472–621 pg/ml, controls: 533 pg/ml, range 237–836 pg/ml) (Figure 2A). However, after exclusion of the edentulous patients in the PLS group (median 909 pg/ml, range 496–2050 pg/ml), significantly higher concentration of MPO (p < 0.05) was found in the PLS saliva than in the saliva from other groups.

In GCF, results were similar, the highest levels of MPO were also measured in samples derived from PLS patients (median 7.50 ng/μl, range 1.95-13.90 ng/μl), followed by chronic periodontitis patients, aggressive periodontitis, and periodontally healthy controls, respectively (Figure 2B). Nevertheless, no significant difference was observed between any groups of samples.

### α-Defensins

The levels of α-defensins in saliva of PLS patients (median 0.33 μg/ml, range 0.00-6.58 μg/ml) were not strikingly different from those in saliva of AP patients and controls (AP: median 0.25 μg/ml, range 0.00-0.5 μg/ml, and controls: median 0.45 μg/ml, range 0.00-6.6 μg/ml). In contrast, chronic periodontitis patients possessed relatively high concentration of HNP1-3 in saliva (median 3.62 μg/ml, range 0.12-19.76 μg/ml) (Figure 2C).

In contrast to saliva, HNP1-3 were detected in GCF from PLS patients (median: 28.98 μg/μl, range: 0.14-103.94 μg/μl) in significantly higher concentration in comparison with periodontally healthy controls (median: 1.20 μg/μl, range: 0–29.19 μg/μl). Only moderate amounts of HNP1-3 were detected in samples from aggressive periodontitis (median: 15.29 μg/μl, range: 0.36-49.61 μg/μl). In chronic periodontitis patients however, the levels of HNP1-3 were high (median: 50.63 μg/μl, range: 0.40-116.17 μg/μl) (Figure 2D). Significantly, both in PLS patients and combined periodontitis patients (AP + CP) the levels of MPO correlated well with amount of detected HNP1-3 (PLS: r = 0.742, p = 0.035; CP + AP: r = 0.533, p = 0.004).

### hCAP18 and cathelicidin LL-37

**Figure 3:**
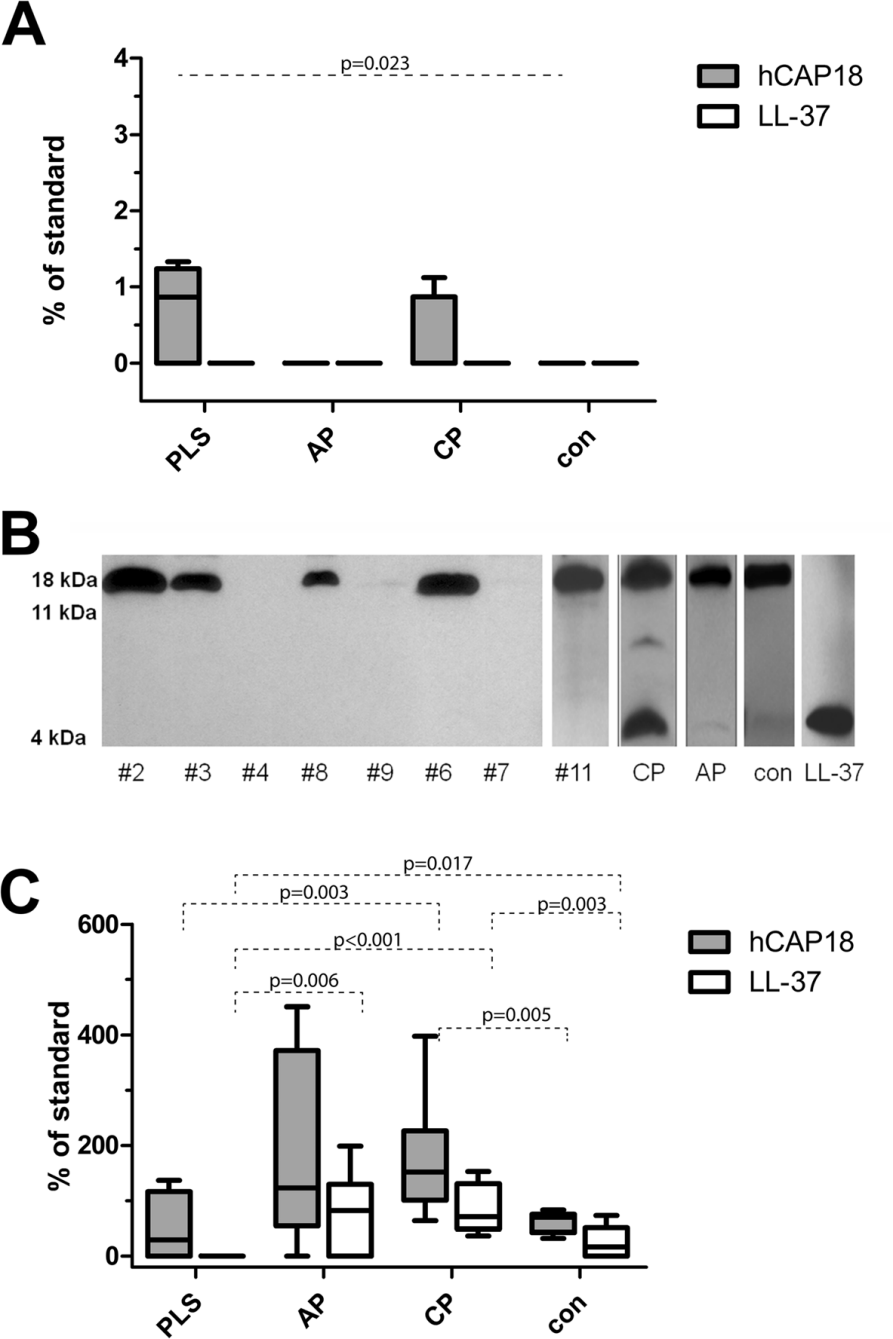
**Levels of unprocessed cathelicidin hCAP18 and mature LL-37 in saliva and gingival crevicular fluid.** Levels of unprocessed cathelicidin hCAP18 and mature LL-37 in saliva **(A)** and gingival crevicular fluid **(C)** obtained from Papillon-Lefèvre patients (PLS), aggressive periodontitis patients (AP), chronic periodontitis patients (CP) and healthy controls (con) determined by western blot analysis. The results present median, and 25^th^ and 75^th^ percentiles. Panel **B** shows Western blot patterns of LL-37 immunoreactive proteins/peptides in GCF samples collected from PLS patients (#1 - #11) as well as representative samples of chronic periodontitis (CP) and aggressive periodontitis (AP) as well as from healthy controls (con). Arrows point to bands immunoreactive against anti-LL-37 antibody, representing intact hCAP18/LL-37, 11 kDa fragment of hCAP18/LL-37, and the LL-37 peptide.

Except for edentulous PLS patients, the unprocessed cathelicidin hCAP18 was always detected in saliva. hCAP18 was present in highest levels in PLS, followed by chronic periodontitis patients and in low abundance in AP patients’ and healthy control individuals’ saliva (Figure 3A). This probably reflects the relatively high level of neutrophils in saliva from PLS and chronic periodontitis groups (Figure 1). The lack of hCAP18 in saliva of edentulous PLS patients correlates with the near absence of neutrophils as indicated by very low amount of MPO in saliva from these individuals. This finding suggests that the gingival crevice around teeth is a major port of entry for neutrophils into the oral cavity. But using Western blot analysis with mAb anti-LL-37 we failed to detect mature LL-37 peptide in any saliva samples regardless of disease status.

High levels of hCAP18, comparable to that of CP, AP and healthy controls, were detected in five (out of eight) analysed samples of GCF from the PLS patients. In three remaining samples barely visible amounts of hCAP18 were found (Figure 3B). In general, the estimated amount of hCAP18 in PLS patients was comparable to that of healthy subjects but lower than in GCF from AP and CP patients (Figure 3C).

Although the LL-37 peptide was found in all GCF samples from periodontally healthy controls, the level of the peptide in CP patients was significantly higher (Figure 3C). In the later group the amount of hCAP18 correlated with mature LL-37 (r = 0.523, p < 0.001) and the load of *P. gingivalis* (r = 0.644, p < 0.001). In AP patients LL-37 was detected at concentrations higher than in controls but the difference was not significant. In strike contrast we failed to detect the mature LL-37 peptide in any of GCF samples of PLS patients despite in part the abundant presence of the precursor protein (Figure 3B). Interestingly in this group the level of hCAP18 showed a correlation to the load of *A. actinomycetemcomitans* (r = 0.793, p = 0.019). Of note, the lack of LL-37 in these samples was not due to proteolytic degradation of the peptide since the externally added peptide was intact in GCF samples even after 24 h incubation (data not shown).

### Activities of cathepsin C, neutrophil elastase and proteinase 3

**Figure 4:**
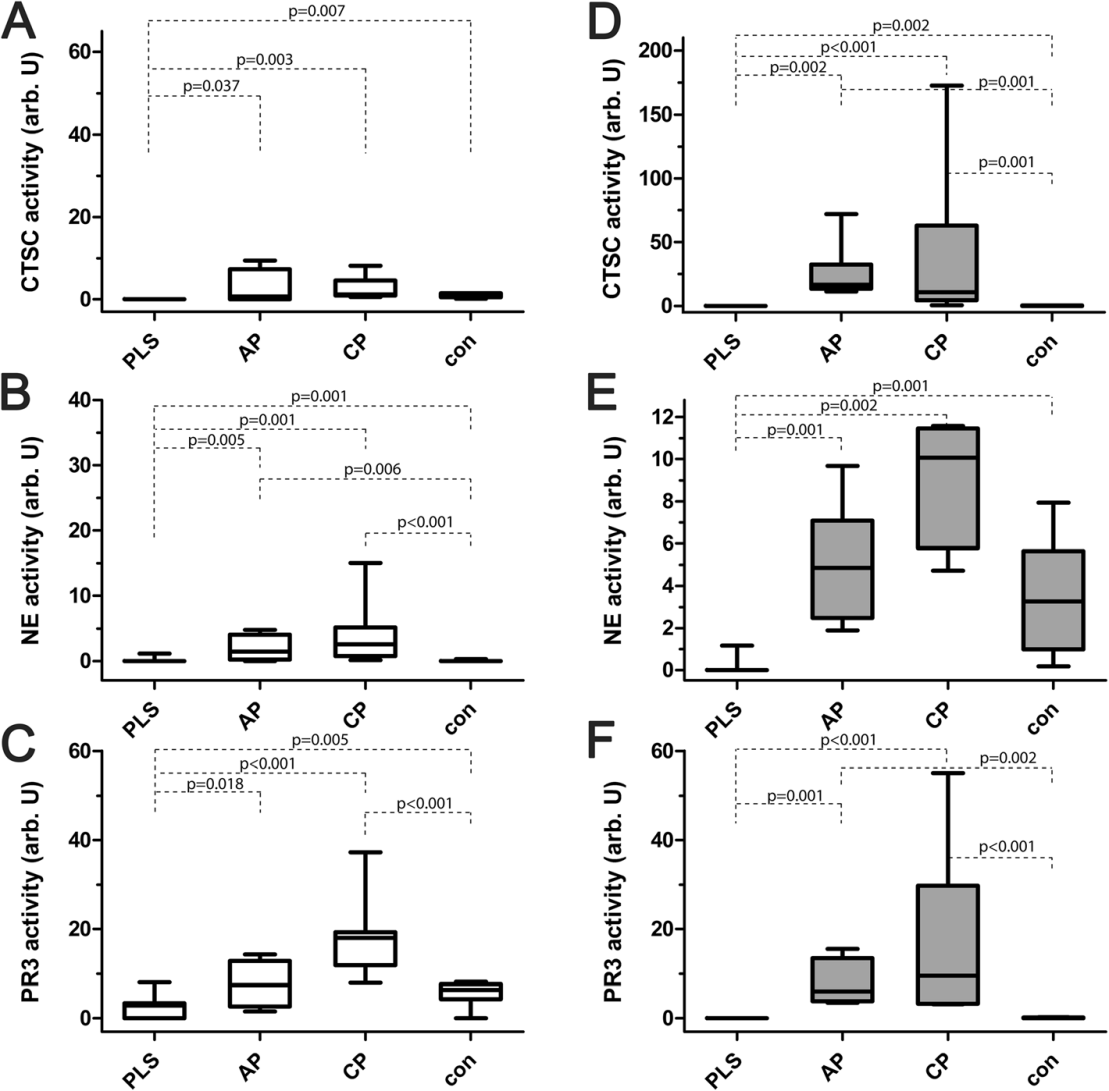
**Activities of cathepsin C, neutrophil elastase and proteinase 3 in saliva and gingival crevicular fluid.** Activities of cathepsin C (CTSC) **(A and** **D)**, neutrophil elastase (NE) **(B and** **D)** and PR3 **(C and** **E)** in saliva (left panels) gingival crevicular fluid (right panels) obtained from Papillon-Lefèvre patients (PLS), aggressive periodontitis patients (AP), chronic periodontitis patients (CP) and healthy controls (con). The results present median, and 25^th^ and 75^th^ percentiles.

We failed to find any CTSC activity in nine out of 11 PLS saliva samples. In all other samples CTSC activity was detected but at highly variable levels. In patients with CP and AP the CTSC activity was slightly higher, but not significantly, compared to periodontally healthy controls (Figure 4A). The lack of the CTSC activity coincided in PLS patients with very low levels of the NE activity comparable to that in saliva from periodontally healthy subjects where the enzyme activity was detected in two out of nine analysed samples. In contrast, the NE activity was high in chronic (CP versus PLS and controls: p < 0.001) and aggressive periodontitis patients (AP versus PLS and controls: p = 0.005 and p = 0.006, respectively) (Figure 4B).

The PR3 activity was detected in six out of the 11 PLS saliva samples (among them in two with the measurable CTSC activity) but at much lower level than in other groups. With exception of one periodontally healthy subject the PR3 activity was present in all other samples (Figure 4C). Significantly, no PR3 activities were detected in saliva of the edentulous PLS patients in keeping with the low level of MPO. This further supports the conclusion that the junction or pocket epithelium around teeth is the most important gate for neutrophils migration into the otherwise healthy oral cavity.

In GCF, the activity of CTSC varied considerably between the groups (p < 0.001). In five samples of PLS patients the CTSC activity was absent while in three other samples it was present at very low levels (Figure 4D). This correlated with the lack of the NE (Figure 4E) and PR3 activities (Figure 4F). In general, the CTSC activity in GCF from PLS was far below that in periodontally healthy subjects (p = 0.002). Conversely, in CP and AP groups the CTSC activity was significantly higher than in healthy controls (AP 103-fold: p = 0.001; CP 66-fold: p = 0.004) (Figure 4D).

### Activity of LL-37 against *Aggregatibacter actinomycetemcomitans* strains

With the exception for one clinical isolate of serotype c, which was resistant, all other tested strains were moderately susceptible to bactericidal activity of LL-37 (Table 2). The resistance does not seem to correlate with the strain serotype since three other strains of the same serotype were sensitive.

**Table 2:** **Minimal bactericidal concentrations (MBC) of LL-37 against** ***Aggregatibacter actinomycetemcomitans*** **strains**

***A. actinomycetemcomitans*** **strain**	**Origin**	**MBC**_**50**_ **(μg/ml)**	**MBC**_**90**_ **(μg/ml)**
ATCC 33384	Laboratory strain	50	70
J1	Clinical isolate	20	70
J2	Clinical isolate	10	50
J3	Clinical isolate	20	50
J7	Clinical isolate	10	50
J11	Clinical isolate	70	>150
J76	Clinical isolate	20	70

MBC_50_ and MBC_90_ indicate the minimal bactericidal concentration (MBC) values at which 50% and 90% of bacterial cells were killed, respectively.

## Discussion

PLS is caused by different mutations in the CTSC gene resulting in typical and atypical pathological outcomes, including those with isolated keratosis and periodontitis [5,24]. Here 11 patients among them nine with typical signs of PLS, including palmoplantar hyperkeratosis and severe early onset periodontitis already in the deciduous dentition in some patients, have been analysed. One patient (#2) had periodontitis of the deciduous dentition, but only suffered from mild skin symptoms. Another PLS patient (#7) suffered from typical skin symptoms, however, exhibited late onset of periodontitis (Additional file 1: Table S1). In all cases we have shown that mutations affected the CTSC activity, never exceeded 10% of the average activity of systemically healthy subjects. Furthermore, we confirmed the observation by Cagli et al. [30] that the expression of dysfunctional CTSC correlated with severely decreased activity of PR3, NE and cathepsin G in neutrophils.

In oral fluids such as saliva and GCF from PLS subjects, the CTSC activity was most absent or detected at very low levels in comparison to the activity in other periodontitis patients and even the periodontally healthy subjects. Deficiency of the CTSC activity in tested fluids was associated with the absence or severe reduction of activity of neutrophil serine proteases, such as NE and PR3. Thus, the inflammatory response to pathogenic bacteria must be disturbed in PLS patients since neutrophil serine proteases activate gingival fibroblasts to produce inflammatory cytokines [31] and are components of neutrophil extracellular traps which trap and kill pathogens [32]. Further, reactive oxygen species generation and MPO activity is not sufficient to kill microbes and proteases are primarily responsible for the destruction of phagocytosed bacteria [33]. This antibacterial activity is exerted in different manners. For example, NE cleaves outer membrane proteins in Gram negative bacteria [34]. In keeping, it was shown that neutrophils from peripheral blood of PLS patients were incapable of neutralizing leukotoxin produced by *A. actinomycetemcomitans* in the process dependent on serine proteases [18].

In stark contrast to saliva samples from PLS patients half of which contain the low but detectable PR3 activity, no trace of the PR3 activity was detected in GCF. Interestingly, in four cases the PR3 activity was found in saliva totally deficient of the CTSC activity. This suggests a possible activation of PR3 by other proteases present in saliva. This is in keeping with results of a study using a leukaemia cell line which indicated that cathepsin C is not the sole enzyme involved in post-translational processing of PR3 [35]. The results implicated PR3 as a promising screening parameter for detection of periodontitis because the PR3 activity was significantly higher in every periodontitis patient without known genetic disorder than in any periodontally healthy control. This conclusion is supported by the recent finding that the PR3-like activity was increased in saliva of periodontitis patients and correlated with severity of the disease [36]. The missing activity in GCF of PLS patients can be an additional hint for checking genetic disorders in selected periodontitis patients. PR3 seems to be an important player in the process of inflammation since it activates oral epithelial cells to produce interleukin-8 and monocyte chemoattractant protein as well as to express intracellular adhesion molecule (ICAM)-1 [37]. This might be in accordance to findings that IL-8 levels were lower in GCF of PLS patients in comparison with controls [38]. In addition, chemotaxis of PMNs to IL-8 is diminished in PLS [39]. However in GCF of PLS patients, neutrophils are present in abundant numbers and this is associated with high levels of α-defensins, other important neutrophil derived antimicrobial peptides. But the used method does not allow distinguishing between the HNP1 precursor and functional HNPs. Considering the postulated role of neutrophil serine protease in processing and activation of HNP-1 [40], the lack of their activity in PLS neutrophils may also cause the deficiency of active HNP1 in periodontal lesions of PSL patients.

Our results indicate that the lack of active neutrophil serine proteases and mature LL-37 is associated with severity of periodontal disease in PLS patients. Recently blood of PLS patients was analysed for LL-37 also using the WB method [18]. In contrast to our finding of the total absence of the peptide in GCF samples, a low amount of mature LL-37 and intermediate-size hCAP18-derived fragments were detected in this study. We found intermediate hCAP18 fragments in chronic periodontitis associated with a high prevalence of *P. gingivalis*, *T. forsythia* and *T. denticola* [27], but never in PLS patients. This discrepancy may arise from different sources of analysed material: isolated neutrophils activated *in vitro* to degranulate versus clinical samples in which neutrophils were *in vivo* exposed to bacteria.

All patients were initially colonized by *A. actinomycetemcomitans,* after receiving an extensive periodontal treatment still four of eight were tested positively for that species, however at low counts. This corroborates with a high prevalence of *A. actinomycetemcomitans* in PLS patients found in other studies [20,41]. Recently by using a 16S rRNA-based microarray *A. actinomycetemcomitans* was found among the species in medium to high levels in 13 untreated PLS patients [42]. Our data strongly suggests that the lack LL-37 is a condition selectively supporting growth of *A. actinomycetemcomitans*, the bacterium directly linked to development and progression of aggressive periodontitis. The importance of *A. actinomycetemcomitans* in PLS patients is underlined by findings of high IgG titers against that species [41,43] and the fact that a successful treatment of localized prepubertal periodontitis in PLS correlates with eradication of *A. actinomycetemcomitans* [20,21].

Interestingly, only in PLS patients the level of unprocessed hCAP18 was correlated with the load of *A. actinomycetemcomitans.* No such correlation between hCAP18 and any periodontal pathogen was found in other groups clearly due to hCAP18 processing and LL-37 release. In these patients LL-37 in conjunction with α- and β-defensins may prevent robust proliferation of *A. actinomycetemcomitans* generating conditions favouring the growth of other periodontal pathogens, including *P. gingivalis*, *T. forsythia* and *P. intermedia*. Indeed, in periodontitis patients without known genetic disorders the load of *P. gingivalis* and the other proteolytic periodontopathogens is correlated with activity of CTSC, NE and PR3 and the released α-defensins and mature LL-37. Data regarding the susceptibility of *A. actinomycetemcomitans* to the various antibacterial peptides are rare, strains were totally insensitive to HNP1-3 [44,45] and show a good to moderate sensitivity to LL-37 [46,47] which was confirmed by our clinical isolates.

Additionally to the missing direct antimicrobial activity a disturbance of other functions modulated by LL-37 may be suggested in PLS patients. These include, direct chemotaxis of immune cells, induction of chemokines, regulation of chemokine receptor expression, inhibition of the release of pro-inflammatory mediators, suppression of neutrophil apoptosis, modification of dendritic cells differentiation and protection against inflammatory shock (reviewed in [48]). *E.g*., LL-37 neutralizes the lipopolysaccharide activity of certain periodontopathogens among them *A. actinomycetemcomitans* in human oral fibroblasts [49]. Although stimulating in low concentrations proliferation of peripheral blood monocytes LL-37 inhibits *in vitro* generation of osteoclasts from these cells [50]. These numerous immunomodulatory activities of LL-37 are apparently essential for maintaining homeostasis in periodontal tissues by providing protective anti-microbial responses without the excessive harmful inflammation.

## Conclusions

In summary, the lack of functional cathepsin C impairs activation of neutrophil serine proteases in neutrophils responding to bacterial challenge in periodontium of PLS.patients. One consequence is the loss of antimicrobial and immunomodulatory functions of LL-37 in the gingiva. This supports infection with *A. actinomycetemcomitans* and the development of severe periodontal disease.

## Additional file

Additional file 1: Table S1. Characteristics (year of birth, ethnicity, sex, phenotype) of the 11 PLS patients including results of microbiological analysis for detection of *Aggregatibacter actinomycetemcomitans* (A.a.) before treatment.
