# Supplementary material for: Lack of cathelicidin processing in Papillon-Lefèvre syndrome patients reveals essential role of LL-37 in periodontal homeostasis
Source: Orphanet J Rare Dis. 2014 Sep 27;9:148. doi: 10.1186/s13023-014-0148-y (PMC4181722; doi:10.1186/s13023-014-0148-y)
Supplement: Additional file 1: Table S1. — Characteristics (year of birth, ethnicity, sex, phenotype) of the 11 PLS patients including results of microbiological analysis for detection of Aggregatibacter actinomycetemcomitans (A.a.) before treatment. [file 13023_2014_148_MOESM1_ESM.doc]

Table S1

Characteristics (year of birth, ethnicity, sex, phenotype) of the 11 PLS patients including results of microbiological analysis for detection of *Aggregatibacter actinomycetemcomitans* (A.a.) before treatment

| **Patient #** | **Year of birth** | **Origin** | **Gender** | **Con-sanguinity** | **Phenotype** | **Nucleotide** | **Exon** | **Effect** | **A.a. positive before / after treatment** | **Blood** | **Saliva** | **GCF** |
| --- | --- | --- | --- | --- | --- | --- | --- | --- | --- | --- | --- | --- |
| 1 *(*6)* | 1979 | Morocco | Male | Yes | PLS (typical findings) | c.854 C>T | 6 | P285L | Yes | X | X | (eden-tulous) |
| 2 (brother of #1) *(*6)* | 1995 | Morocco | Male | Yes | PLS (mild skin findings) | c.854 C>T | 6 | P285L | Yes / Yes | X | X | X |
| 3 *(*5,6)* | 1999 | Eritrea | Male | No | PLS (typical findings) | c.755 A>T | 5 | Q252L | Yes / Yes | X | X | X |
| 4 (brother of #3) *(*5,6)* | 2002 | Eritrea | Male | No | PLS (typical findings) | c.755 A>T | 5 | Q252L | Yes / No | X | X | X |
| 5 *(*1,3,6)* | 1988 | Germany | Female | Yes | PLS (typical findings) | c.566-572Del | 4 | T189FS199X | Yes / Yes | X | X | X |
| 6 (sister of #5)  *(*1,3,6)* | 1991 | Germany | Female | Yes | PLS (typical findings) | c.566-572Del | 4 | T189FS199X | Yes / Yes | X | X | X |
| 7 *(*6)* | 1966 | Germany | Male | No | PLS (late onset of periodontitis at the age of 22) | c.322 A>T  c.436delT  (compound heterozygous) | 3 | K108X  S146fs153x | Unknown | X | X | (eden-tulous) |
| 8 *(*2,4,6)* | 1987 | Germany | Male | No | PLS (typical findings) | c.947 T> G  c.1268 G>C  (compound heterozygous) | 7 | L316R  W423S | Yes / No | X | X | X |
| 9 *(*6)* | 2000 | Russia | Male | Adopted | PLS (typical findings) | c.566-572Del  c.628C>T  (compound heterozygous) | 4 | T189FS199X  R210X | Yes / No | X | X | X |
| 10 *(*6)* | 2001 | Germany | Male | No | PLS (typical findings) |  |  |  | Yes / unknown | X | X | (not available) |
| 11 | 1971 | Hungary | Male | No | PLS (typical findings) |  |  |  | Unknown / No | X | X | X |

**Previously reported cases:*

*1 Rüdiger et al. 1999*

*2 Eickholz et al. 2001*

*3 Hewitt et al. 2004*

*4 Noack et al. 2004*

*5 Schacher et al. 2006*

*6 Noack et al. 2008*
